# Polymeric Biomaterials for the Treatment of Cardiac Post-Infarction Injuries

**DOI:** 10.3390/pharmaceutics13071038

**Published:** 2021-07-07

**Authors:** Sonia Trombino, Federica Curcio, Roberta Cassano, Manuela Curcio, Giuseppe Cirillo, Francesca Iemma

**Affiliations:** Department of Pharmacy, Health and Nutritional Sciences, University of Calabria, 87036 Rende, CS, Italy; sonia.trombino@unical.it (S.T.); federica.curcio@unical.it (F.C.); giuseppe.cirillo@unical.it (G.C.); francesca.iemma@unical.it (F.I.)

**Keywords:** cardiac regeneration, tissue engineering, biomaterials, hydrogels, nanoparticles

## Abstract

Cardiac regeneration aims to reconstruct the heart contractile mass, preventing the organ from a progressive functional deterioration, by delivering pro-regenerative cells, drugs, or growth factors to the site of injury. In recent years, scientific research focused the attention on tissue engineering for the regeneration of cardiac infarct tissue, and biomaterials able to anatomically and physiologically adapt to the heart muscle have been proposed as valuable tools for this purpose, providing the cells with the stimuli necessary to initiate a complete regenerative process. An ideal biomaterial for cardiac tissue regeneration should have a positive influence on the biomechanical, biochemical, and biological properties of tissues and cells; perfectly reflect the morphology and functionality of the native myocardium; and be mechanically stable, with a suitable thickness. Among others, engineered hydrogels, three-dimensional polymeric systems made from synthetic and natural biomaterials, have attracted much interest for cardiac post-infarction therapy. In addition, biocompatible nanosystems, and polymeric nanoparticles in particular, have been explored in preclinical studies as drug delivery and tissue engineering platforms for the treatment of cardiovascular diseases. This review focused on the most employed natural and synthetic biomaterials in cardiac regeneration, paying particular attention to the contribution of Italian research groups in this field, the fabrication techniques, and the current status of the clinical trials.

## 1. Introduction

Myocardial infarction (MI) represents one of the leading causes of morbidity worldwide, with a mortality rate of 17.9 million people per year [1]. The main limitation to the proper recovery of myocardial functionality after a heart injury lies in the modest endogenous capability to regenerate the damaged tissue, which is usually replaced with unfunctional connective tissue [2]. Thus, there is tremendous interest in finding permanent solutions to restore the cardiac functionality while attenuating tissue remodeling and fibrosis [3,4].

The available therapeutic strategies are designed to target one of the five main processes associated with MI, namely, massive cardiomyocyte death [5], inflammation [6], remodeling of the extracellular matrix (ECM) [7], angiogenesis [8], and cardiomyogenesis [9]. In detail, the prevention of cardiomyocyte death can be achieved by either repression of apoptotic processes or stimulation of survival pathways; manipulation of the chemokine/cytokine profile or cellular responses during the inflammation process, which can help in modulating a proper tissue repair; modulation of the balance between the matrix metalloproteinases and tissue residing factors, which can avoid scar formation, thus favoring a desired tissue healing; stimulation of pro-angiogenesis signals, which can stimulate the formation of new blood vessels; and, finally, induction of the proliferation/transplantation of cardiomyocytes, which is a key requirement for the full restoration of cardiac function [10,11,12].

Cell-based therapy, consisting of the direct injection of an autologous or heterologous cell suspension into the myocardium, is a suitable strategy for repairing or replacing injured cardiac tissue [13]. Human embryonic stem cells (hESC) and induced pluripotent stem cells (iPSC) have been recognized as valuable tools for this purpose, because of their ability to effectively differentiate into cardiomyocytes [14,15]. On the other hand, this therapeutic approach suffers for some drawbacks related to the need to inject a great number of cells due to the low cell survival and poor retention rate (almost 10%). Furthermore, for achieving a proper differentiation into cardiomyocytes and organization of the regenerated tissue, the presence of both biochemical signaling and mechanical support, acting as topographical guidance, is strongly required [16,17].

Cardiac tissue engineering, coupled with regenerative medicine, represents a very useful approach to repair or regenerate damaged tissues or organs and restoring their functions. In the last decades, due to the possibility to design scaffolds with tailored physic-chemical and biomechanical features, biomimetic devices based on synthetic or natural polymers have attracted much interest in this field [18,19]. The design of a performing biomaterial takes advantage of the collaboration of researchers belonging to different scientific areas, from chemistry, physics, and engineering, to technology, biology, and medicine. Depending on the application area, the properties of the final biomaterials can be tailored by selecting the most appropriate polymer, as well as the synthetic and formulation processes [20], which are the core expertise of Pharmaceutical Technology. Cardiac scaffolds based on natural or synthetic biomaterial can mimic the ECM environment, with the further possibility to combine the cell therapy with the release of bioactive molecules [21]. In this regard, different kinds of systems, either in the form of injectable or implanted scaffolds, have been proposed to date [22,23].

In this review, we aim to overview the recent advances in the development of biomaterials for cardiac tissue regeneration, paying particular attention to the contribution of Italian research groups in this field. The referenced works were classified according to the nature of the base materials (organic or inorganic, natural, or synthetic), highlighting the adopted synthetic strategy and the main outcomes.

## 2. Biomaterials for Cardiac Regeneration

Any biomaterial designed for cardiac tissue engineering should possess key properties to prevent the dilation of cardiac muscle, avoid or slow scar formation and fibrosis, while favoring the integration and proliferation of cardiomyocytes [24].

At first, high biocompatibility and non-immunogenicity are required to avoid adverse effects during the healing process [25]. For any kind of in vivo application, an ideal biomaterial should produce a beneficial or neutral response upon interaction with the host tissue or environmental components at the site of application. In the case of cardiac applications, apart from the interaction with all the components of the myocardium (e.g., cardiomyocytes, endothelium, fibroblasts, and perivascular cells) and the compatibility of the metabolic by-products, the blood–material interaction is also a big challenge, since the material’s exposure to the blood flow can result in thrombosis or embolism events [25].

The immune response triggered by an implanted biomaterial can results in either positive (tissue regeneration with angiogenesis, remodeling, and restoration of functionality) or negative (tissue repair with fibrosis and scar formation) healing effects [26]; thus, the induction of an immune response favoring tissue regeneration is critical for successful treatments. Although the base mechanisms eliciting a regenerative response are not fully understood, experimental evidence allows hypothesizing that surface chemistry and degradation rates are pivotal parameters [27]. The kinetics of scaffold degradation should be ideally close to that of new tissue formation, to guarantee adequate space availability for the newly formed tissue and effective regeneration before the scaffold is completely degraded. A fast degradation rate can result in incomplete host infiltration, further compromising the organ integrity; on the other hand, inhibition of cell remodeling and angiogenesis, together with scar formation, is the main phenomenon accompanying the overly long persistence of the scaffold [28].

Furthermore, an ideal scaffold should possess a porosity degree in the range 50–90%, to promote the diffusion of nutrients, oxygen, and extracellular fluids through the cellular networks—mechanical properties allowing the mechanical strength of the organ to be retained until complete regeneration of heart tissue—as well as the right balance between stiffness and flexibility to support repeated stretch cycles, without constraining the contractions and relaxation of cardiac muscle [25].

**Figure 1:**
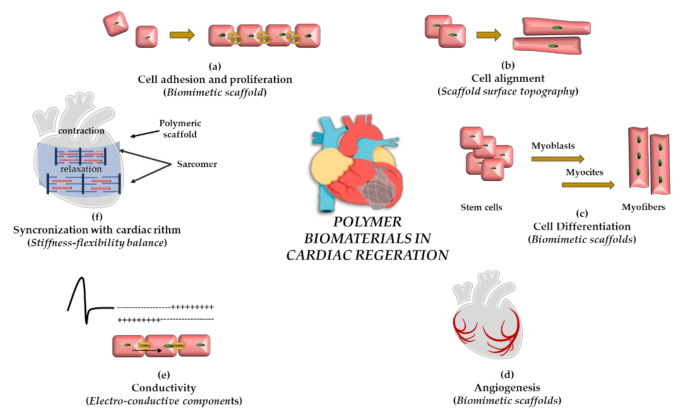
Representation of the biological effects required for myocardial regeneration induced by polymeric biomaterials. (**a**) Increased cell proliferation and adhesion; (**b**) greater cell alignment; (**c**) cell differentiation; (**d**) induction of angiogenesis; (**e**) cellular electrical coupling and enhanced impulse conduction; (**f**) synchronization with cardiac rhythm.

As schematically depicted in Figure 1, to promote cell engraftment and reorganization into a 3D tissue, the cardiac scaffold should match the key features of the heart, as well as the unique interplay between the cardiac cells and native cardiac ECM components [29]. It should possess tailored mechanical properties, such as anisotropy, elasticity, and contractility, to provide the required pressure for an effective pump function while withstanding the tensile stress [30]. The surface properties should be tailored with functional niches to provide the cells with anchoring positions promoting attachment, anisotropic alignment, and proliferation [31]. Thus, scaffold stiffness and geometry are important features to be considered, with square pores being more efficient in promoting cell adhesion rather than hexagonal pores [32]. Moreover, biochemical cues, such as growth factors [33], cytokines, and adhesion molecules, should be inserted to induce effective maturation of cardiac cells [21]. Finally, in order to produce the optimal material for tissue interfaces, the design of the cardiac biomaterial should fit with the complex electrical pathways of the myocardium [34], obtained by a fine coordination between membrane potential depolarization, pacemaker conduction system, and specific intracellular communication networks [35].

Ultimately, since an ideal cardiac patch should possess features close to that of the cardiac ECM, some clinical trials aimed to investigate the safety and effectiveness of the cardiac patches based on a natural ECM [1]. The ECM cardiac patch CorMatrix^®^ (CorMatrix Cardiovascular, Inc. Vascular surgeon in Roswell, GA, USA) was claimed to promote endogenous cardiac regeneration, although trials were able to prove its safety rather than its effectiveness (ClinicalTrials.gov identifier: NCT02887768) [36]. More encouraging results were obtained with VentriGel^TM^ (Ventrix, Inc., San Diego, CA, USA), a porcine-cardiac ECM-injectable hydrogel, which was found to be safe and improves exercise capacity (ClinicalTrials.gov identifier: NCT02305602) [37].

Among the great number of materials available for the fabrication of cardiac scaffolds mimicking the ECM functionalities, polymers from either natural or synthetic origin well match with the above-described requirements.

Natural polymers, such as polysaccharides and proteins, possess many favorable properties, including biocompatibility and biodegradability, and have been applied as scaffold in cardiac regeneration because of their similarity with the natural tissues and the ability to facilitate cell adhesion, proliferation, and differentiation [38]. In addition, the presence of many functional groups allows the polymer backbone to be easily functionalized, and the physic-chemical properties to be finely tuned [39]. On the other hand, synthetic polymers are versatile materials with high porosity, durability, and physic-chemical and structural properties, capable to fit with the particular needs of the target tissue [40]. In most cases, to overcome the limitations of each class of polymer, while enhancing their strengths, natural and synthetic polymers can be combined into composite materials with superior properties [41]. Following the same rationale, inorganic or organic/inorganic hybrid scaffolds were also proposed, in order to mimic either the main inorganic components of natural tissues or their intrinsic properties [42]. For example, considering the electrical properties of native myocardium, conductive fillers can be introduced, facilitating the electrical coupling between adjacent cells within the scaffolds [35]. Organic, inorganic, and hybrid biomaterials can be formulated into different types of constructs, which, for our convenience, are here classified as injectable hydrogels, cardiac patches, and nanoparticles.

Injectable hydrogels, three-dimensional hydrophilic polymeric networks with a high water content, are formed by in situ gelation and represent very advantageous devices for tissue engineering and drug delivery [43]. These formulations can be injected directly into the site of interest with a syringe without surgery, enhancing the patient compliance, with the cells loaded into the hydrogel during the gelation process [44,45]. Specifically, for cardiac applications, hydrogels should possess proper mechanical stiffness, as well as the ability to retain cells after transplantation while providing them with a suitable physical and biochemical microenvironment for proliferation [46,47,48]. A cardiac patch is defined as a piece of in vitro-grown, functioning heart tissue on an engineered support that can replace part of the injured tissue. The cells can be cultured on, or suspended into, the biomaterial matrix, obtaining cell sheets and cell-containing scaffolds, respectively [49].

Finally, nanoparticle systems are well known for their ability to enhance the pharmacokinetics profiles of any bioactive molecules and are often used as delivery systems to facilitate the healing process of the cardiac tissues [50].

## 3. Cardiac Scaffolds Fabrication Techniques

Since the scaffolds’ effectiveness strictly depends on their properties, it is evident that the choosing the proper fabrication method is a key determining factor. The available techniques for the fabrication of cardiac scaffolds can be divided into conventional and non-conventional methods. The first group includes solvent casting/particulate leaching, thermally-induced phase separation, electrospinning, gas foaming, and freeze drying [24,51].

In the solvent casting/particulate leaching method, the selected polymeric materials are dissolved in a highly volatile solvent in the presence of a suitable porogen (e.g., water-soluble inorganic salts or sugars) and poured into a mold. After solvent evaporation, porogens are leached out by immersing the composite in water with formation of the porous scaffold [52]. In this technique, the porogen amount influences the scaffold porosity, while pore size and geometry can be modulated by varying the particulate size and shape [53].

Thermally induced phase separation is a versatile technique where temperature variation induces a phase separation of a polymer solution into low and high polymer concentration phases [54]. In a typical procedure, the selected polymer is dissolved at a high temperature in a solvent with a low melting point, and the porous scaffold is obtained after cooling below the solvent melting (liquid–liquid phase separation) or solidification (solid–liquid phase separation) points. In the first case, the solvent is then removed under vacuum, while in the solid–liquid phase separation, solvent crystals are removed by washing with a non-solvent of the polymer and then applying a vacuum-drying or freeze-drying procedure. Pore size is influenced by the cooling temperature and solvent crystallization for liquid–liquid and solid–liquid phase separation, respectively, with the heat transfer conditions greatly influencing the geometry [55,56].

Electrospinning involves the application of a strong electric field (10–20 kV) on a polymer solution placed in a needle [57]. Under these conditions, the polymer solution is charged, and flows at a controlled rate to a collector put at a specific distance from the needle. While moving, the solvent evaporates, with the subsequent deposition of the polymeric fibers with mean diameters in the range of 3 nm to 5 μm [58]. The fiber morphology can be tuned to the varying ambient conditions, such as temperature, humidity, and air velocity, as well as to the instrumental parameters (e.g., applied voltage, flow rate, needle size, needle-to-collector distance, and collector shape and composition). Moreover, to generate functional scaffolds, the solution properties, such as viscosity, conductivity, surface tension, and polymer molecular weight, should be optimized [59].

In the gas-foaming procedure, polymer discs are firstly exposed to supercritical CO_2_, and then the pressure is quickly decreased to atmospheric, leading to the formation of clusters of thermodynamically unstable CO_2_ into the polymer structures, and thus pore nucleation occurs. Under these conditions, high porous scaffolds can be obtained, although with the disadvantages of poor pore interconnectivity and the formation of a nonporous layer surface [60].

The freeze-drying process is an advantageous technique allowing the obtainment of porous scaffolds without using high temperatures and avoiding any washing step to remove the porogen. Following this technique, a frozen polymer solution, poured in a mold, is freeze-dried under vacuum, obtaining materials with a porosity depending on the pH and freezing rate [61].

The conventional techniques suffer from some disadvantages related to the difficulty in controlling the scaffold architecture and mimicking the ECM structure, and are often poorly reproducible. Thus, so-called unconventional techniques have been proposed, including three-dimensional (3D) printing, laser ablation, and pressure-assisted microsyringe.

3D printing possesses great potential in producing scaffolds for biomedical applications [62]. The procedure, based on a bottom-up approach, involves a computer-assisted combination of materials in 3D shape. Among the different 3D printing techniques, Selective Laser Sintering (SLS) is the most used for biomedical applications [63]. In SLS, polymer particles are locally fused together into solid structures by the application of a high-powered laser (infrared or CO_2_ laser), with the motion of the laser beam being controlled by a computer-aided platform. A layer-to-layer overlay allows highly complex and tailored scaffolds to be formed [64]. The method is cost effective due to the possibility to recycle the unused particles, and highly versatile since it allows tuning scaffolds properties by modulating the processing parameters, such as the particle size, laser power, and scan speed [65].

Different natural and synthetic polymers can be shaped into 3D cardiac scaffolds by laser ablation, consisting of the thermal or photochemical removal of materials from bulk [66], although the high energy required in the process limits the application of this methodology.

Finally, a well-defined scaffold geometry can be obtained by the pressure-assisted microsyringe technique, where a 5–20 μm capillary needle is used to extrude a polymer dissolved into a high volatile solvent. The viscosity of the polymer solution, together with the needle diameter and the applied pressure, contributes to determine the scaffold’s morphology and surface properties [67].

## 4. Natural Polymers in Cardiac Regeneration

Natural polymers are ideal candidates for the preparation of cardiac scaffolds, as documented by an analysis of the current clinical trials in the field [1].

Italian research in this field mainly involves the use of collagen (COL), gelatin (GEL), and silk fibroin (FIB) as the protein materials, while chitosan (CHI), alginate (ALG), heparin (HEP), and hyaluronic acid (HYA) represent the most explored polysaccharides. The main examples of biomaterials based on natural polymers proposed for cardiac regeneration are shown in Table 1.

COL is the major fibrous protein of the ECM, composed of tropocollagen monomers, formed by three left-handed polypeptide chains rich in glycine, proline, and hydroxyproline, that join to form a triple right helix [81]. Among the 28 types of collagen, type I is the main component of ECM myocardium and the most investigated in cardiac tissue engineering [82], because of its ability to mimic the native cardiac structure, promoting tissue formation, cell differentiation in vitro, and the maintenance of myocardial geometry during the cardiac cycle [83,84]. The potential of type I collagen sponge in promoting the neovascularization process was tested both in in vitro and in vivo studies. The collagen scaffolds were applied on the epicardial surface of both cryoinjured and intact rat hearts, finding the complete absorption of the collagen scaffold after 60 days post-injury time and the appearance of new arterioles and capillaries in both experimental models [68].

**Figure 2:**
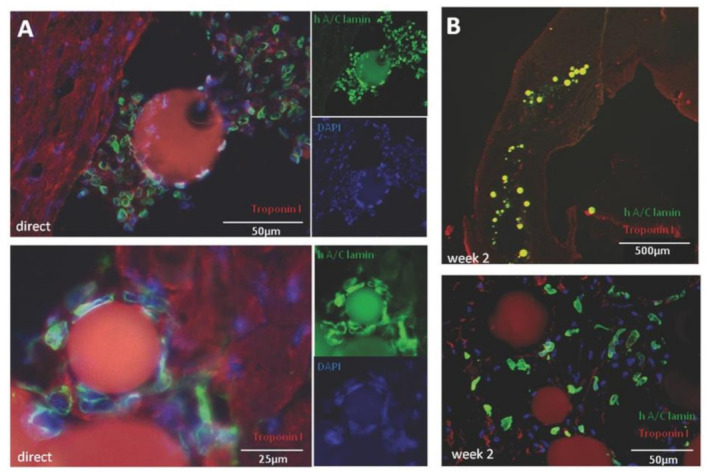
Injection of CPC-laden gelatin MS into murine myocardium. (**A**) Histological analysis. (**B**) Two weeks post-injection. Reproduced with permission from [69], 2016, John Wiley and Sons.

GEL, the hydrolysis product of collagen, was also used as base materials for cardiac tissue engineering. Gelatin microspheres with a dimensional range of 50–75 μm were prepared by the water-in-oil emulsion process in the presence of glutaraldehyde as crosslinker and proposed as microcarriers of human cardiac progenitor cells (CPC) in the ischemic myocardium. The microparticles were able to promote in vitro cell attachment, maintaining their cardiogenic potential, while in vivo models of myocardial infarction revealed that the significant increase in cell engraftment in myocardial tissue was not accompanied by an equal improvement in cardiac function, compared to CPC only [69] (Figure 2).

Silk-based materials, such as films, hydrogels, nano- and micro-nets, and sponges [85,86], are a promising class of systems for biomedical application due to their tunable mechanical features, biodegradation, and biocompatibility. Biomaterials based on silk fibroin, obtained by directly treating degummed fibers, have been employed to induce the regeneration of various mammalian tissues, including bone, cartilage, tendon, and skin [87,88].

FIB scaffolds with three different geometries (two sponges with different pore sizes and distributions and an electrospun nanometric net) were prepared and the influence on CPC differentiation and integrin, cardiac, and sarcomere protein expression evaluated in vitro [70]. The study demonstrated that the scaffolds embedded with a collagen-containing medium and seeded with CPC can efficiently drive cell commitment, as demonstrated by the high level of sarcomere and cardiac proteins, as well as by the great quantity of ECM observed after 21 days.

Fibrinogen and its enzymatic hydrolysis product, fibrin (FBR), are glycoproteins extensively employed in cardiac tissue engineering by virtue of their peculiar characteristics, such as an improved healing mechanism, protective against myocardial reperfusion injury, and increased cell migration [89,90,91]. In the literature, there have been reports on the application of fibrin matrices loaded with mesenchymal stem cells [92] and human embryonic stem cells [93] as valuable scaffold materials able to improve left ventricle contraction and prevent heart failure. An FBR cardiac patch was involved in a clinical trial (ClinicalTrials.gov identifier: NCT02057900). Here, cardiac progenitors embedded in the patch were epicardially delivered during a coronary artery bypass procedure. The results showed the ability of the patch to promote the formation of highly purified cardiac progenitor cells, without the occurrence of arrhythmia phenomena [94].

CHI is another key material explored for cardiac applications because of either its sustainability (it is a by-product of the food industry), biocompatibility, biodegradability, and antibacterial properties, or its cationic nature, promoting the interaction with the anionic glycosaminoglycans and proteoglycans of the cardiac ECM [95]. The latter is a key determining factor for cardiac applications, since the presence of glycosaminoglycan in the cardiac ECM is a critical factor for modulating the functionalities of specific proteins, such as growth factors [96]. Moreover, the high versatility of CHI allows the fabrication of scaffolds with a tunable morphology and chemo-physical properties, including the porosity and pore level [97,98]. A wide range of 3D-printed CHI scaffolds [99,100] have been proposed for skin [101] and bone [102] regeneration, while thin scaffolds crosslinked by either genipin or epichlorohydrin with two different oriented porosities were employed as cardiac patches [71]. The possibility to modulate the available chemical functionalities (e.g., amino and hydroxyl groups for epichlorohydrin and genipin, respectively), together with that to control the porosity and the micro-channel orientation, were considered as the added value of the proposed scaffolds, since the regeneration of cardiac tissue requires a preferential cell orientation.

Saporito et al. have developed an injectable in situ gelling systems based on an anionic polysaccharide, such as ALG, and on chondroitin sulphate (CHS) loaded with platelet lysate (PL), to improve the survival rate of the cardiomyocytes after myocardial infarction. The choice of ALG as base material for repairing myocardial infarction, cardiac regeneration, supporting heart vascularization, re-cellularization, and restoring electrical conductivity [103], is related to its ability to form high viscous solutions due to long-range interactions within the polymer chains [104], thus allowing the formation of hydrogels in mild conditions. The results of in vitro studies on fetal heart cells showed that the system is able to maintain a prolonged residence time of the PL, allowing a high degree of cardiac cells survival (either cardiomyocytes or cardiac fibroblasts) after oxidative damage [72]. Bloise et al. hypothesized that the activation of immune-mediated mechanisms of heart repair by the release of a colony-stimulating factor and anti-inflammatory interleukins 4/6/13 from a Ca^2+^ crosslinked ALG hydrogel can stimulate wound healing and restore myocardial function after the infarct [73].

The suitability of ALG injectable hydrogels for cardiac regeneration was proved by three different clinical trials. A bio-absorbable ALG hydrogel (IK-5001, Bellerophon BCM LLC) injected into the infarct-related artery was found to be totally safe, even if the treatment did not reduce heart remodeling (ClinicalTrials.gov identifier: NCT01226563) [105]. On the other hand, a significant improvement in cardiac function parameters (e.g., ejection fraction, end-systolic and diastolic volumes, and average wall thickness) was recorded after treatment with acellular ALG hydrogels (Algisyl-LVR^TM^, LoneStar Heart Inc., (Mission Viejo, CA, USA) ClinicalTrials.gov identifier: NCT0084796) [106]. The same hydrogel was also tested on 78 patients with dilated cardiomyopathy, obtaining a remarkable amelioration of the exercise capacity (ClinicalTrials.gov identifier: NCT01311791) [47].

HYA, a fundamental component of the ECM, is involved in cellular proliferation and differentiation, and thus in many biological processes, including wound repair and inflammation [107]. Muscari et al. investigated the transplantation of autologous mesenchymal stem cells (MSC) with a hyaluronan-based knitted scaffold to restore the functionality of the infarcted myocardium via induction of neo-angiogenesis and histological modifications of the cardiac cells [74]. The advantage of inserting HYA in a cardiac scaffold originates from its role in angiogenesis and inflammation modulation, thus allowing neovascularization of cardiac tissue and increasing the capillary density and normalizing left ventricular function in an infarcted heart [108]. Contrast-enhanced ultrasound studies showed that the native tissue interacted positively with the scaffold by modifying the extracellular matrix with a reduced presence of collagen and an increased content of proteoglycans. As a result, a lower degree of cardiomyocyte damage was observed, with the absence of any trace of inflammatory process at the infarct site, probably due to the action of the grafted MSC attenuating cellular infiltration.

As before mentioned, proteins and polypeptides were often combined with polysaccharides in order to obtain composite systems mimicking the ECM composition and able to promote cell division and growth during the tissue regeneration process [109].

A blend of COL and CHI, obtained by electrophoretic deposition, was proposed as a highly biocompatible 3D-scaffold to support fibroblast as well as cardiomyocyte adhesion and proliferation. The attachment, spreading, and orientation of cardiomyocytes were affected by the blend of the composition, showing the suitability of the proposed material for promoting cardiac tissue regeneration [75].

The water-in-oil emulsion method was employed to fabricate GEL-GLL microparticles as injectable scaffolds to repair the infarcted myocardium. The systems showed good injectability and persistence at the injection site. Moreover, by in vitro cell culture assays, the influence of particle diameter on cardiac progenitor cells was demonstrated, indicating a preferential cell adherence to microparticles with a smaller size [76]. After loading with insulin-like growth factor-1 (IGF-1) by absorption, in vivo experiments were performed, finding an attenuated chamber dilatation and myocardial damage and fibrosis, together with an improved cell homing [77].

ALG was used as a polysaccharide counterpart in the synthesis of GEL- or COL-based blends crosslinked both by Ca^2+^ ions and by glutaraldehyde for cardiac regeneration [78]. Different ALG-to-GEL weight ratios were tested and the best results in terms of cell proliferation using C2C12 myoblasts were obtained for the blends containing more than 60% GEL, with the ALG/GEL ratio of 20:80 showing the ability to promote cell differentiation [79].

Heart patches based on GEL and CHS were prepared by means of electrospinning and proposed as fibrous implants to improve heart recovery after corrective surgery for critical congenital heart defects. CHS improved the mechanical properties of the system, increasing the elasticity and reducing the stiffness. The nanofibrous scaffolds were loaded with PL as a source of growth factors to enhance the tissue repair. Interestingly, the patches appeared to selectively favor the proliferation of cardiomyocytes rather than cardiac fibroblasts, thus reducing the fibrosis phenomena [80]. A 3D-printed biocomplex, composed of a HYA/GEL matrix system and CPC, was transplanted into a mouse model of myocardial infarction, leading to a significant reduction in adverse remodeling and preservation of cardiac performance, as evidenced by both magnetic resonance imaging and histology. In addition, the matrix supported the long-term in vivo survival and engraftment of CPC, which exhibited a temporal increase in cardiac and vascular differentiation markers over the course of the 4-week follow-up period.

## 5. Synthetic Polymers in Cardiac Regeneration

Several biodegradable polymers, such polyethylene glycol (PEG), poly(εcaprolactone) (PCL), poly(glycerol sebacate) (PGS), poly(l-lactide) (PLA), poly(lactic-co-glycolic acid) (PLGA), biodegradable polyurethanes (PUR), and poly(butylene succinate) (PBS), gained considerable interest for cardiac regeneration [110] (Table 2).

To promote cardiac cell alignment, PGS and PCL were combined in patterned electrospun fibers, simulating the architecture of cardiac tissue. Since nano- and micro-scale topographical features of the fibers play a critical role in the induction and maintenance of various cellular properties and functions, different surface topographies were investigated, such as squares and grooves, with constant or different interspatial distances. The results of in vitro cell culture assays showed that the surface topography influenced the cardiomyocytes’ orientation and morphology, without affecting the cells’ viability [111]. Sub-micrometric fibers of poly(ω-pentadecalactone) obtained by electrospinning were proposed as biocompatible scaffolds characterized by long degradation times. The adhesion and proliferation of rat cardiac H9C2 cells on the scaffold surface was evaluated, verifying that the cells retained their morphology and form a confluent monolayer [112]. In a more recent work, a series of random PBS-based copolymers containing PEG-like sequences of triethylene succinate (TES) was synthesized by melt polycondensation and tested as scaffolds for embryonic rat cardiac H9C2 cell adhesion and proliferation and as delivery devices of the anti-inflammatory drug dexamethasone (DMT). It was found that by varying the molar percentage of TES in the copolymer, the thermal and mechanical properties, surface wettability, and hydrolysis rate of the material can be easily modified. In addition, the proposed biomaterials showed good biocompatibility properties, with the copolymers containing up to 20 mol% of TES co-units, sustaining a better cell adhesion and proliferation as well as the highest encapsulation capability and the fastest DMT release kinetics [113].

Among the different types of synthetic polymers used for the preparation of cardiac devices, PUR shows high versatility, because of the biocompatibility and the elastic properties necessary to avoid plastic deformation or failure, and the large number of macrodiols, diisocyanates, and chain extenders available for the preparation. Silvestri et al. reported on the synthesis of four biodegradable scaffolds by thermally-induced phase separation, adding poly(ester urethanes) and poly(ether ester urethanes) from PCL and PEG as macrodiols, 1,4-diisocyanatobutane as a diisocyanate, L-lysine ethyl ester, and alanine–alanine–lysine peptide as chain extenders, to confer enzymatic degradability to the final system. Elastase degradation tests demonstrated the possibility to finely tune the biodegradation rate, while the best results in terms of cell proliferation were obtained with the scaffold containing the lowest amount of PEG, probably for the more adequate microstructure favoring cell attachment [114]. PUR bi-layered scaffolds, endowed with elastomeric-like behavior, were prepared by melt-extrusion additive manufacturing using PCL as the diol, BDI as the diisocyanate, and L-lysine ethyl ester dihydrochloride as the chain extender. The resulting scaffolds were found to efficiently support CPC adhesion and spreading, although a poor proliferation was observed after a 1–14 days culture time [115]. The same PUR system was functionalized by plasma-mediated grafting with laminin-1 (LN1), a protein protecting CPC from apoptosis and stimulating their proliferation [52]. Compared to pristine PUR and the system obtained using GEL as the functionalizing protein, the resulting PUR-LN1 scaffold showed an increased cell density, an improved cell protection against apoptosis, and enhanced CPC proliferation, stimulating their differentiation into cardiomyocytes, endothelial cells, and smooth muscle cells [51].

In order to investigate the influence of scaffold anisotropy and ECM incorporation on the pathological remodeling process initiated by myocardial infarction, three different microfibrous, biodegradable patches composed of poly(ester carbonate urethane)urea were prepared. The results showed that the bi-layered patch containing ECM promoted angiogenesis, inhibiting scar formation and left ventricle wall thinning, typical key phenomena of maladaptive remodeling following myocardial infarction. On the other hand, the patches with the stiffer direction parallel to the heart circumferential direction (orthogonal group) and with the longitudinal direction of the heart (longitudinal group) did not produce significant effects on echocardiographic function, wall thinning, or scar formation [116].

Electrically conductive polyurethane/siloxane networks, containing different amounts of aniline tetramer (AT) as conductive moieties, were proposed for cardiac tissue engineering application. Castor oil was employed as the biodegradable source of polyols whereas siloxane domains guaranteed mechanical strength properties. The results of the biological experiments demonstrated that AT affects the attachment and proliferation of C2C12 myoblasts, confirming the potential application of the materials as a cardiac patch [117].

Polylactic acid (PLA) derives from polycondensation of lactic acid, a monomer of natural origin produced by the bacterial fermentation of carbohydrates, or by ring opening polymerization of the cyclic dimer lactide [125]. PLA can be easily obtained in the form of fibers, films, and sheets that can be used not only as scaffolds for tissue regeneration but also as drug delivery vehicles [126,127]. In cardiac tissue engineering, PLA is widely used because it is a cytocompatible and biodegradable material with different physical-chemical, mechanical, and thermal properties, depending on the stereo-regularities obtained from the L or D enantiomers of lactic acid during polymerization [128,129]. Moreover, it is often combined with other synthetic polymers in order to obtain materials with optimized biomechanical properties [130].

A commercial copolymer of (L)-lactic acid with trimethylene carbonate (PLA-co-TMC), well known for its interesting thermal, mechanical, and degradation behaviors, was employed as starting material to obtain a biomimetic electrospun scaffold, and its performance compared with a PLA homopolymer scaffold. PLA-co-TMC modified its mechanical properties by varying the surrounding temperature, being a glassy rigid material at room temperature and a rubber-like soft material at 37 °C, and when seeded with cardiomyocytes, efficiently promoted cell proliferation, preserving cell morphology [118]. A PLA electrospun scaffold releasing granulocyte colony-stimulating factor (GCSF) was tested as a ventricular patch in a rabbit chronic model of myocardial infarction. It was found that the scaffold efficiently integrated into a chronic infarcted myocardium, and that the functionalization of the biopolymer with GCSF led to increased fibroblast-like vimentin-positive cellular colonization and reduced inflammatory cell infiltration within the micrometric fiber mesh in comparison to nonfunctionalized scaffold. Moreover, a PLLA/GCSF polymer induced an angiogenetic process with a statistically significant increase in the number of neovessels compared to the nonfunctionalized scaffold and, when implanted at the infarcted zone, induced a reorganization of the ECM architecture, leading to connective tissue deposition and scar remodeling. These findings were combined with a reduction in end-systolic and end-diastolic volumes, indicating a preventive effect of the scaffold on ventricular dilation, and an improvement in cardiac performance [119].

The main limitation in the use of PLA is its degradation products, in particular, lactic acid, which is a relatively strong acid that can cause an inflammatory response when it accumulates internally, accelerating the degradation of the implant and the loss of its mechanical integrity. Thus, different approaches have been proposed to mitigate the shortcomings of this synthetic polymer, including the preparation of a copolymer of lactic acid and glycolic acid: poly(lactic-co-glycolic acid) (PLGA) [131]. PLGA microparticles were used as a carrier of growth factors to simulate cell proliferation and organization in vessels [132]. When applied in cardiac regeneration, PLGA microparticles loaded with human adipose-derived stem cells, hepatocyte growth factor, and IGF-1 were incorporated into an injectable hydrogel, to obtain a multifunctional system able to stimulate the survival and/or differentiation of the grafted cells toward a cardiac phenotype. Following the growth factors release, an enhanced synthesis of cardiac differentiation markers was observed, as well as an accelerated cell cycle progression. Moreover, the addition of the thermosensitive P407 hydrogel to the microparticulate system led to an improvement of its elasticity properties and an increase in junction connections in human adipose-derived stem cells [120].

Between the many biopolymer-based scaffolds already studied, poly(vinyl alcohol) (PVA) devices have a great potential for cardiac tissue engineering because of their excellent mechanical properties, such as stability and flexibility, conferring an adaptive behavior towards the strain changes caused by cardiac contractions [133]. Moreover, PVA retain a significant amount of water or biological fluids, swelling without dissolving. Dattola et al. reported on the fabrication of a biocompatible, porous PVA scaffold, employing a combination of gas-foaming and freeze-drying processes, avoiding any cross-linking agents. An analysis of the stress–strain curves proved that the obtained scaffolds were characterized by an elastic behavior similar to that of the muscle ECM, while the potential applicability as a cardiac device was confirmed by the ability to support human-induced pluripotent stem cell growth and differentiation into cardiomyocytes [121].

**Figure 3:**
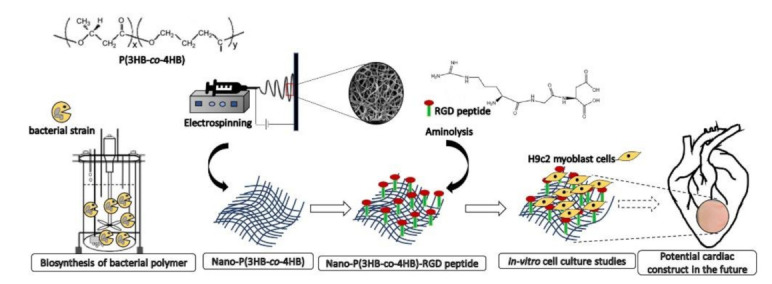
Schematic representation of the nano-P(3HB-co-4HB) scaffold. Reproduced from [123], 2020, Frontiers.

A useful approach to stimulate cell activity is the surface conjugation of scaffolds with the RGD (R: arginine; G: glycine; D: aspartic acid) peptide, a well-established cell adhesion motif. RGD is a tri-amino acid constituting a variety of ECM proteins, playing a pivotal role in the regulatory functions of many biological activities [134]. In an interesting work, Burgess et al. reports on an alternative approach to promote the delivery and retention of cardiac progenitor cells into the heart based on an injectable self-assembling peptide hydrogel functionalized with RGD. The results of the study demonstrated that CPC cultured in vitro within the hydrogel spontaneously differentiated towards adult cardiac for one-week phenotypes. Furthermore, after injection, the hydrogel on its own resulted in a reduction in heart damage, and when loaded with CPC, was able to retain them for up to 10 days at the injury site, with a significant reduction in left ventricular dilation [122]. The RGD peptide was immobilized by aminolysis on a scaffold based on poly(3-hydroxybutyrate-co-4-hydroxybutyrate) [P(3HB-co-4HB)], a bacterial copolymer with desirable mechanical and physical properties, demonstrating that the surface modification enhanced the hydrophilicity of the system, promoted the cell–scaffold interaction, and enhanced the attachment and proliferation of H9C2 myoblast cells [123] (Figure 3).

Finally, non-degradable polyacrylate copolymers were proposed as useful materials in the valve scaffold manufacturing process. The interest towards this class of copolymer is derived from their highly tunable mechanical properties, high biocompatibility, and the possibility to be functionalized with various biologics, such as adhesion molecules, binding repeats, extracellular matrix components, and cytokines. Poly(methoxyethylmethacrylate-co-diethylaminoethylmethacrylate) was used as a coating agent of non-woven PCL scaffold and seeded with valve interstitial cells (VIC). The authors observed that VIC increased the secretion of the elastin-maturing component and of other valve-specific extracellular matrix components, proving the effectiveness of the proposed valve implants in promoting VIC adherence and growth, and avoiding their evolution toward a pro-calcific phenotype [124].

## 6. Composite and Hybrid Systems in Cardiac Applications

An ideal scaffold for cardiac regeneration should be highly biocompatible, support cell proliferation, and possess adequate elasticity to not compromise heart contractile function. To fully satisfy these requirements, composite and hybrid systems are often proposed, to combine the key properties of the individual components [135,136] (Table 3).

### 6.1. Composite Materials

Pure fibrinogen or fibrin scaffolds often suffer from some limitations related to their poor mechanical properties that may restrict their field of use [153]. Thus, fibrinogen or fibrin were combined with synthetic polymers in order to reinforce the whole structure, improve the elasticity, and the resistance to deformation forces [154].

Poly(ethylene glycol)-fibrinogen scaffolds were loaded with iPSC cells bioengineered to secrete placental growth factor and matrix metalloproteinase 9 factors involved in vascularization and engraftment processes. When injected in infarcted rat myocardium, an improved revascularization and hemodynamic parameters were recorded, together with an interesting functional integration of allograft-derived cells and host myocardium [137]. Hydrogels based on FIB and polyethylene-glycol-diacrylated were prepared by photopolymerization and proposed as sponge-like scaffolds and injectable carriers for cardiac mesenchymal stem cell differentiation. Bovine serum albumin microspheres were added to the hydrogel system to increase the porosity and cell-adhesive properties of the material. Good viability and expression of proteins characteristic of the initial phases of the cardiac muscle differentiation process were recorded, with a further increase in the cell viability after the addition of chondroitin sulfate into the scaffolds [138].

In another work, enzyme-coated bovine serum albumin microbubbles able to catalyze the H_2_S release were loaded into a polyethylene glycol–fibrinogen hydrogel in order to favor the proliferation and differentiation of human Sca-1 pos cardiac progenitor cells (hCPC), with micropores within the scaffold serving as biological cues to promote cell attachment and maintain cell morphology. The authors claimed the possibility to employ this H_2_S-releasing 3D scaffolds to minimize the ischemic effects and reperfusion damages in the implant site thanks to the properties of the H_2_S [139]. In addition, FBR layers were reinforced with poly(ether)urethane–polydimethylsiloxane, obtaining a semi-interpenetrating polymeric network with desirable elastic properties and able to sustain growth and differentiation of human amniotic mesenchymal stromal cells [140].

The combination of an ALG/GEL blend with poly(N-isopropylacrylamide)-co-2-hydroxyethylmethacrylate-6-hydroxyhexanoate by the micromolding technique carried out to the fabrication of 3D scaffolds with cardiac ECM-like microarchitecture. Due to their favorable properties, such as superficial microporosity, elastic behavior, and anisotropic properties similar to that of an adult human left ventricular myocardium, the materials were proposed as effective scaffolds for myoblast proliferation and differentiation [141]. In order to reinforce the performances of the 20:80 ALG/GEL sponge blend and improve the suturing properties of the final system [78], fibrous structures based on polycaprolactone (PCL) were inserted. The resulting material perfectly combined the hydrophilicity of the natural blend and the mechanical properties of the synthetic polymer without compromising the biocompatibility [142].

Polydioxanone was also investigated as a reinforcing agent of an ALG/GEL blend functionalized with IGF-1, using the avidin–biotin-binding strategy, with the in vitro and in vivo biological characterization demonstrating the enhanced cell adhesion and long-term retention after implantation on the damaged myocardium, together with an improved suture ability [143].

A PLGA/GEL-based biomaterial with controlled degradation kinetics was proposed as a scaffold for human MSC. The system, mimicking the anisotropic structure and the mechanical properties of cardiac tissue, has found to promote adhesion, long-term viability, and ordered disposition of MSC, as a confirmation of its suitability in restoration of myocardium viability [144]. Ciuffreda et al. proposed a hydrogel system based on PEG and acrylated heparin (HEP) as a synthetic substitute for the extracellular matrix, with the aim to couple the well-known treatments of ischemic heart disease by MSC transplantation with the ability of heparinized biomaterials to stimulate angiogenesis in both subcutaneous implants and wound healing models. The authors found a significant increase in MSC engraftment, a reduction in ventricular remodeling, stimulation of neo-vasculogenesis, and an increase in several pro-angiogenic factors [145]. Zuluga et al. proposed the delivery of Astaxanthin, an FDA approved xanthophyll carotenoid derivative, by a composite hydrogel consisting of polyvinyl alcohol/dextran/cyclodextrins. Astaxanthin allows the mitigation of oxidative stress in the heart by blocking ROS and reducing the myofibril stress [146].

### 6.2. Hybrid and Inorganic Materials

In order to improve the performance of the cardiac scaffolds in terms of mechanical, electrical, and functional properties, inorganic counterparts were combined to natural or synthetic polymers to obtain hybrid systems with superior features.

The work of Scalera et al. was designed to address the limitation of the low electro-conductivity of CHI-based materials [147]. In detail, due to the well-known possibility to improve the conductivity of different types of biomaterials by incorporation of carbon nanostructures [155], the authors focused their attention on the production of graphitic materials from natural sources, and from pyrolyzed cork in particular. The investigation of the scaffold properties clearly proved the enhanced conductivity and mechanical properties of CHI materials upon incorporation of inorganic carbon, without affecting the high biocompatibility and the degradation patterns. Interestingly, a sample containing 1% (by weight) of carbon filler was found to possess electro-conductivity close to that of cardiac muscle, while the pyrolyzed cork is intended to match with the objectives of the European Green Deal.

Another therapeutic approach is the mitigation of the oxidative stress in the infarcted heart. In this regard, Hao et al. explored the possibility to use fullerenol nanoparticles loaded into an injectable ALG hydrogel network as an antioxidant vehicle for cell release [156]. The presence of fullerenol was found to be able to scavenge superoxide anions and hydroxyl radicals, thus ensuring cardiomyogenic differentiation of brown adipose tissue. Single-walled carbon nanotubes (SWCNT) were embedded in a GEL crosslinked matrix and used as scaffolds for the H9C2 cell line. By varying the SWNCT amount, the mechanical, electrical, and biological behaviors of the hybrid system could be tuned, while the phenotypical changes in the H9C2 cell line was evaluated by modifying the culture medium composition. In particular, the best result in terms of cell differentiation was obtained with fetal bovine serum and all-trans retinoic acid concentrations of 1% and 50 nm, respectively [148]. Hybrid injectable biomimetic scaffolds were obtained by the covalent functionalization of lysine-derivatized poly(serinol hexamethylene urea)-co-poly(N-isopropylacrylamide) (RGT-lysine) with multi-walled carbon nanotubes (MWCNT). The copolymer guaranteed a sol-gel phase transition near to body temperature, ideal for biomedical applications, whereas the MWCNT improved the rheological and electrical properties. The results of the biological assays suggested that the chemical conjugation of MWCNT to RGT-lysine enhanced the biocompatibility of the system, with the obtained hybrid scaffold showing an improved long-term cardiac cell survival (up to 21 d), proliferation, and function compared to the same material obtained in absence of MWCNT [149].

Magnetic nanoparticles (MNPs) were also employed as inorganic counterparts of PLA to obtain ultrathin nanofilms. Since the MNP content affected the roughness and wettability of the scaffold, different materials were prepared and the effect of the surface properties on cytocompatibility, adhesion, proliferation, and differentiation of H9C2 evaluated. It was found that the MNPs did not compromise cell viability, improving their adhesion, proliferation, and differentiation properties [150].

Totally inorganic nanoparticles were investigated as scaffolds or delivery systems of bioactive materials into cardiac tissue. Due to the similarity with the main inorganic component of bones, teeth, and some pathological calcification, calcium phosphate nanoparticles were proposed as a promising platform for in vitro and in vivo miRNA delivery in polarized tissue, such as the heart. The advantage of using this kind of material lies in their high biocompatibility and pH-sensitive stability, allowing for the complete release of their payload in biological acidic environments, such as endosomes and lysosomes [151]. In another approach, the ability of silica nanoparticles (SiO_2_-NP) to facilitate stem cell adhesion capability on cardiac tissue was investigated. SiO_2_-NP treatment increases the surface expression of the gap junctions on MSC, increasing the intercellular communications with cardiomyoblasts in an ischemia-like environment [152].

## 7. Conclusions and Perspectives

Irreversible damage to cardiac tissue after myocardial infarction is the main cause of a progressive loss of organ function that often evolves into heart failure and death. Cardiac tissue regeneration based on biomaterial scaffolds represents an emerging therapeutic strategy to repair the injured heart and improve heart function. Natural or synthetic polymers have attracted much interest for the development of biocompatible supports able to promote the direct transplantation of cells into the injured environment, act as replacement tissues, stimulate the organ in the regeneration of damaged tissues through direct administration of growth factors, and ensure the implantation of polymeric supports able to recruit and stimulate the patient’s own cells. This review paper covers the broad range of polymeric materials used for cardiac regeneration purposes developed by the Italian scientific community, highlighting the formulation strategies as well as main outcome results.

The majority of studies dealt with the evaluation of cardiac scaffolds and patches as cell delivering systems, promoting stem cell proliferation and differentiation. The high similarity with the ECM makes the polysaccharides/protein composites the most widely proposed starting materials, with most studies covering alginate and gelatin derivatives. Furthermore, synthetic polymers and inorganic nanoparticles are often added to the natural system to modulate the mechanical and rheological properties and confer electrical conductivity, respectively. The outcomes of the studies are at a preliminary but promising stage, underlining the huge interest in the field, as well as that a more exhaustive and critical evaluation of the preclinical data is required before setting up clinical trials. The main issues are the mechanical stability, immunogenic responses, and a proper integration within the host myocardium. A possible answer to these challenges could be the design of a cardiac patch able to treat the harmful consequences of MI and, at the same time, to restore proper cardiac functionalities by delivering specific biological cues.

It should be also underlined that the results of the current clinical trials demonstrated the safety of the proposed patched, while not fully showing their effectiveness in terms of cardiac regeneration, as recorded in the preclinical studies. Clearly, the presence of a gap between the preclinical studies and clinical applications could be filled by the proper exchange of information and synergy between scientists working in different research areas, including technologists, engineers, and clinicians. Only by matching the biomedical requirements with tailored formulation protocols would it be possible to provide insight and valuable strategies that might lead to the lab-bench-to-clinic translation of cardiac biomaterials.

## Figures and Tables

**Table 1:** Biomaterials based on natural polymers proposed for cardiac regeneration applications in Italian research.

Composition	Formulation	Preparation	Model		Outcomes		Ref.
			In Vitro	In Vivo	In Vitro	In Vivo	
COL	Patches	Preformed Sponges	SMC HUVEC CM	Wistar rats	Cell growth and differentiation	Angiogenesis Arteriogenesis	[68]
GEL	Microspheres	Water-in-oil emulsion	CPC	NOD SCID mice	Cell engraftment	Cell accumulation	[69]
FIB	Scaffolds	Freeze-drying Electrospinning	CPC	-	Overexpression of cardiac proteins and ECM	-	[70]
CHI	Patches	Electrochemical deposition	MS1	-	Biocompatibility	-	[71]
ALG CHS	Injectable hydrogels	In situ gelation	CM CF	-	Cell growth and differentiation	-	[72]
ALG	Hydrogels	Ionic gelation	CD14+	Sprague Dawley rats	Biocompatibility	Enhanced wound healing	[73]
HYA	Scaffolds	Preformed scaffolds	MSC	Swine	Cell growth and differentiation Synthesis of VEGF	Cell growth and differentiation Angiogenesis	[74]
COL/CHI	Scaffolds	Electrophoretic deposition	HFF C2Cl2 CM iPSC	-	Cell adhesion and orientation Cell growth and differentiation	-	[75]
GEL/GLL	Microparticles	Water-in-oil emulsion	CPC Porcine heart	-	Cell adhesion Cell growth	-	[76]
GEL/GLL	Microparticles	Water-in-oil emulsion	CPC	Wistar rats	Cell adhesion Cell growth Release of IGF-1	Cell growth	[77]
GEL/ALG COL/ALG	Sponges	Ionic and chemical gelation	C2C12	-	Cell growth and differentiation	-	[78]
GEL/ALG	Scaffolds	Ionic and chemical gelation	C2C12	-	Cell growth and differentiation	-	[79]
GEL/CHS	Patches	Electrospinning	NHDF HUVEC CF/CM	-	Biocompatibility Cell adhesion Cell growth and differentiation	-	[80]

ALG: alginate; C2C12: mouse myoblast; CD14+: CD14 positive human peripheral blood monocytes; CF: cardiac fibroblasts; CHI: Chitosan; CHS: chondroitin sulfate; CM: cardiomyocytes; COL: collagen; CPC: cardiac progenitor cells; FIB: silk fibroin; GEL: gelatin; GLL: gellan; HFF: human foreskin fibroblasts; HUVEC: human umbilical vein endothelial cells; HYA: hyaluronic acid; IGF-1: insulin-like growth factor 1; iPSC: pluripotent stem cells; MS1: mouse endothelial cells; MSC: mesenchymal stem cells; NHDF: normal human dermal fibroblasts; rCM: rat neonatal cardiomyocytes; SMC: vascular smooth muscle cells; VEGF: vascular endothelial growth factor.

**Table 2:** Biomaterials based on synthetic polymers proposed for cardiac regeneration applications in Italian research.

Composition	Formulation	Preparation	Model		Outcomes		Ref.
			In Vitro	In Vivo	In Vitro	In Vivo	
PGS/PCL	Fibers	Electrospinning/soft lithography	C2C12 rCM	-	Cells orientation and morphology dependent on fibers topography	-	[111]
PPDL	Fibers	Electrospinning	H9C2	-	Cell adhesion and proliferation	-	[112]
P(BSmTESn)	Film	Compression molding	H9C2	-	Cell adhesion and differentiation depending from comonomer ratio	-	[113]
	Nanoparticles	Water-in oil mini-emulsion	DMT release experiments in physiological conditions		Encapsulation and kinetic release depending from comonomer ratio		
PUR	Porous scaffolds	Thermally-Induced Phase Separation	H9C2	-	Cell viability dependent from PUR composition	-	[114]
PUR	Porous scaffolds	Melt-extrusion	CPC	-	Cell adhesion and proliferation	-	[115]
PUR-LN-1	Biomimetic scaffold	Melt-extrusion/carbodiimide chemistry	CPC	FVB Mice	Cell adhesion and proliferation	Angiogenesis	[51]
PUR	Patches	Electrospinning	-	Lewis rats	-	Angiogenesis/Scar formation inhibition/Left ventricle wall thinning inhibition	[116]
PUR/SiO/AT	Film	Sol-gel reaction	C2C12	-	Electro-conductivity/Cell adhesion and proliferation		[117]
PLA-co-TMC	Fibers	Electrospinning	CM	-	Cell proliferation/Morphology preservation	-	[118]
PLA-GCSF	Fibers	Electrospinning	-	Rabbits	-	Angiogenesis/Reorganization of the ECM architecture	[119]
PLGA	Injectable hydrogel	Emulsion solvent extraction-evaporation	ADSC	-	Cell growth and differentiation	-	[120]
PVA	Scaffolds	Gas foaming/freeze drying	iPSC	-	Cell growth and differentiation	-	[121]
Polypeptide-RGD	Injectable hydrogel	Self-assembling	rCPC	Wistar rats	Cell differentiation	Reduced heart damage	[122]
P(3HB-co-4HB)-RGD	Fibers	Electrospinning/aminolysis	H9C2	-	Cell adhesion and proliferation	-	[123]
PMEMA-co-DEAMA-coated PCL	Preformed discs coating	Dip-coating	VIC	-	Cell growth	-	[124]

ADSC: human adipose-derived stem cells; AT: aniline tetramer; C2C12: mouse myoblast; CM: cardiomyocytes; CPC: human cardiac progenitor cells; DMT: dexamethasone; ECM: extracellular matrix; GCSF: granulocyte colony-stimulating factor; H9C2: heart myoblast; iPSC: pluripotent stem cells; LN-1; laminin-1; [P(3HB-co-4HB)]: poly(3-hydroxybutyrate-co-4-hydroxybutyrate); P(BSmTESn): poly(butylene/triethylene succinate); PCL: poly(εcaprolactone); PGS: poly(glycerol sebacate); PLGA: poly(lactic-co-glycolic acid); P(L)LA-co-TMC: polylactide-trimethylcarbonate; PMEMA-co-DEAMA: poly(methoxyethylmethacrylate-co-diethylaminoethylmethacrylate); PPDL: poly(ω-pentadecalactone); PUR: polyurethanes; rCM: rat neonatal cardiomyocytes; rCPC: rat cardiac progenitor cells; PVA: poly(vinyl alcohol); RGD: arginine–glycine–aspartic acid; SiO: siloxane; VIC: valve interstitial cells.

**Table 3:** Composite and hybrid biomaterials for cardiac regeneration applications in Italian research.

Composition	Formulation	Preparation	Model		Outcomes		Ref.
			In Vitro	In Vivo	In Vitro	In Vivo	
PEG-FBN	Patches	Radical Polymerization	iPSC	NOD SCID mice	Cell growth and differentiation	Cell growth and differentiation Angiogenesis	[137]
BSA-MPs@PEG-CHS-FIB	Injectable Hydrogel	Radical Polymerization	CMSC	-	Cell growth and differentiation	-	[138]
BSA-MBs@PEG-FBR	Hydrogel	Radical Polymerization	HFF CPC	-	H_2_S release	-	[139]
PEtU-PDMS/FBR	Hydrogel	Spray phase inversion	AMSC	-	Cell growth and differentiation	-	[140]
PNIPAAm/HEMAHex-ALG/GEL	Scaffolds	Micromolding	C2C12	-	Cell adhesion and growth	-	[141]
ALG/GEL-PCL	Scaffolds	Molding	H9C2	-	Cell growth and differentiation	-	[142]
ALG/GEL-PDO	Scaffolds	Ionic and chemical gelation	CPC	Rat	Cell growth and differentiation	Restoring of cardiac functions	[143]
PLGA/GEL	Scaffolds	Solvent casting	MSC	-	Cell Adhesion and alignment Cell growth and differentiation		[144]
PEG-HEP	Hydrogel	Radical Polymerization	MSC MCS	Sprague Dawley rats	Biocompatibility Angiogenesis	Cell retention and engraftment Cell growth Angiogenesis	[145]
PVA/DEX/βCD	Hydrogel	Molding	3T3 H9C2	-	Biocompatibility Cell growth	-	[146]
CHI/PCP	Scaffolds	Freeze-drying	SH-SY5Y	-	Electrical conductivity Biocompatibility	-	[147]
GEL/SWCNT	Scaffolds	Chemical gelation	H9C2	-	Electrical conductivity Biocompatibility	-	[148]
PSHU−PNIPAAm/MWCNT	Scaffolds	Condensation	NRVM	-	Long term cells survival Cell growth and differentiation	-	[149]
PLA/MNP	Films	Spin-coated assisted deposition	H9C2	-	Biocompatibility Cell adhesionCell growth and differentiation	-	[150]
CaP	Nanoparticles	Precipitation	HL-1 CM	Mice	Biocompatibility	MiRNA delivery	[151]
SiO_2_	Nanoparticles	Water-in-oil microemulsion	MSC	-	Cell adhesion Cell growth and differentiation	-	[152]

3T3: mouse fibroblast; ALG: alginate; AMSC: human amniotic mesenchymal stromal cells; BSA: bovine serum albumin; C2C12: mouse myoblast; CaP: calcium phosphate; CD: cyclodextrin; CHS: chondroitin sulfate; CMSC: cardiac mesenchymal stem cells; CPC: cardiac progenitor cells; DEX: dextran; FBN: fibrinogen; FBR: fibrin; FIB: fibroin; GEL: gelatin; H9C2: rat embryo ventricular cardiomyocytes; HEMAHex: 2-hydroxyethylmethacrylate-6-hydroxyhexanoate; HEP: heparin; HFF: human foreskin fibroblasts; HL-1: cardiac muscle cell line; iPSC: pluripotent stem cells; MBs: microbubbles; MCS: mononuclear cells from Sprague Dawley rats; MNP: magnetic nanoparticles; MPs: microparticles; MSC: mesenchymal stem cells; MWCNT: multi-walled carbon nanotubes; PNIPAAm: poly-N-isopropylacrylamide; NRVM: neonatal rat ventricular myocytes; PCL: polycaprolactone; PCP: pyrolyzed cork powder; PLA: poly(lactic acid); PLGA: poly(lactic-co-glycolic acid); PDMS: polydimethylsiloxane; PDO: polydioxanone; PEG: poly(ethylene glycol); PEtU: poly(ether)urethane; PSHU: poly(serinol hexamethylene urea); PVA: polyvinyl alcohol; SH-SY5Y: neuroblastoma cell line; SHU: serinol hexamethylene urea; SWCNT: single-walled carbon nanotubes.

## Data Availability

Not applicable.
